# Possible mediators of metabolic endotoxemia in women with obesity and women with obesity-diabetes in The Gambia

**DOI:** 10.1038/s41366-022-01193-1

**Published:** 2022-08-06

**Authors:** Modou Jobe, Schadrac C. Agbla, Marijana Todorcevic, Bakary Darboe, Ebrima Danso, Jean-Paul Pais de Barros, Laurent Lagrost, Fredrik Karpe, Andrew M. Prentice

**Affiliations:** 1 Medical Research Council Unit The Gambia at London School of Hygiene & Tropical Medicine, Banjul, The Gambia; 2 Department of Health Data Sciences, University of Liverpool, Liverpool, UK; 3 Department of Infectious Disease Epidemiology, London School of Hygiene & Tropical Medicine, London, UK; 4 Oxford Centre for Diabetes, Endocrinology and Metabolism, Oxford, UK; 5 Plateforme de Lipidomique-uBourgogne, INSERM UMR1231/LabEx LipSTIC, Dijon, France; 6 University Hospital of Dijon, Dijon, France; 7 NIHR Oxford Biomedical Research Centre, OUH Foundation Trust, Oxford, UK

**Keywords:** Type 2 diabetes, Obesity

## Abstract

**Aims/hypothesis:**

Translocation of bacterial debris from the gut causes metabolic endotoxemia (ME) that results in insulin resistance, and may be on the causal pathway to obesity-related type 2 diabetes. To guide interventions against ME we tested two hypothesised mechanisms for lipopolysaccharide (LPS) ingress: a leaky gut and chylomicron-associated transfer following a high-fat meal.

**Methods:**

In lean women (*n* = 48; fat mass index (FMI) 9.6 kg/m^2^), women with obesity (*n* = 62; FMI 23.6 kg/m^2^) and women with obesity-diabetes (*n* = 38; FMI 24.9 kg/m^2^) we used the lactulose-mannitol dual-sugar permeability test (LM ratio) to assess gut integrity. Markers of ME (LPS, EndoCAb IgG and IgM, IL-6, CD14 and lipoprotein binding protein) were assessed at baseline, 2 h and 5 h after a standardised 49 g fat-containing mixed meal. mRNA expression of markers of inflammation, macrophage activation and lipid metabolism were measured in peri-umbilical adipose tissue (AT) biopsies.

**Results:**

The LM ratio did not differ between groups. LPS levels were 57% higher in the obesity-diabetes group (*P* < 0.001), but, contrary to the chylomicron transfer hypothesis, levels significantly declined following the high-fat challenge. EndoCAb IgM was markedly lower in women with obesity and women with obesity-diabetes. mRNA levels of inflammatory markers in adipose tissue were consistent with the prior concept that fat soluble LPS in AT attracts and activates macrophages.

**Conclusions/interpretation:**

Raised levels of LPS and IL-6 in women with obesity-diabetes and evidence of macrophage activation in adipose tissue support the concept of metabolic endotoxemia-mediated inflammation, but we found no evidence for abnormal gut permeability or chylomicron-associated post-prandial translocation of LPS. Instead, the markedly lower EndoCAb IgM levels indicate a failure in sequestration and detoxification.

## Introduction

Metabolic endotoxemia (ME) has been proposed as a possible mechanism to explain the strong linkage between obesity, inflammation, impaired glucose tolerance (IGT) and the development of type 2 diabetes (T2D) [1–3]. In ME, bacterial debris such as lipophilic lipopolysaccharide (LPS) translocates from the gut, homes to adipose tissue, and attracts macrophages [4, 5]. A resultant inflammatory cascade of cytokines and adipokines [6] can induce insulin resistance by, among other possible mechanisms, the phosphorylation of insulin receptor substrate [7].

Our prior work in The Gambia has shown elevated fasting serum LPS levels in people with diabetes compared with those without diabetes [8], supporting and extending work elsewhere [9–11]. We also found that people with obesity and people with obesity and diabetes (henceforth, obesity-diabetes) had much lower levels of endotoxin IgM antibody (EndoCAb) than lean subjects suggesting that rates of LPS ingress are higher than the capacity to sequester and detoxify them [8].

In a healthy gut, the intestinal mucosal epithelium serves as an effective barrier to restrict the egress of microbial products such as LPS by means of a tight epithelial lining, the mucus layer, immunoglobulins, and other antimicrobial agents [12]. The translocation of microbial products across the gut wall of people with obesity and people with obesity-diabetes could have several explanations. Paracellular trans-epithelial leakage is known to occur in a number of conditions where tight junction integrity is compromised leading to a ‘leaky’ gut [13, 14]. Recent work has shown that this might be a downstream consequence of persistent hyperglycaemia rather than being on the causal pathway [15]. Additionally several studies have shown a strong association between LPS translocation and the consumption of high-fat meals either through a chylomicron-associated transfer of LPS [16] or through internalisation by intestinal microfold cells [17].

To discriminate between these possibilities we studied lean women, women with obesity and women with obesity-diabetes in The Gambia to assess: (a) gut permeability; (b) the acute effects of high-fat meals on serum LPS and related immunological and inflammatory biomarkers; and (c) the expression of inflammatory cytokines and macrophage markers in adipose tissue.

## Methods

### Participants

We recruited 3 study groups of Gambian women (25–60 years of age): lean (BMI: <24.9 kg/m^2^), women with obesity (BMI: ≥30 kg/m^2^) and women with obesity-diabetes (BMI: ≥30 kg/m^2^ and with a clinical diagnosis of type 2 diabetes). The first two groups were identified from house-to-house screening in the communities of Bakau and Serrekunda of the Kanifing Municipality in The Gambia. The women with obesity-diabetes were recruited from the diabetes clinic at the Edward Francis Small Teaching Hospital in Banjul. Participants were excluded if they had diarrhoea, acute febrile illness in the preceding 2 weeks, were on renal dialysis, had a known/confirmed history of inflammatory bowel diseases, were receiving treatment for hyperlipidaemia or antibiotics (at the time or for the 2 preceding weeks), or were using laxatives (at the time). Lean women and women with obesity were also screened for diabetes and excluded if found to have diabetes. A trained field assistant provided detailed study information in the participant’s language prior to consenting and recruitment.

Participants’ weight was measured to the nearest 0.1 kg using daily calibrated, digital scales (Tanita Corporation, Tokyo, Japan) and height was measured using a calibrated stadiometer (Leicester height measure, Seca 214, Birmingham, UK) to the nearest 0.1 cm. Body composition was further assessed by bioelectrical impedance analysis using the Tanita BC-418 MA (Tanita Corporation, Tokyo, Japan) segmental analyser. Blood pressure was measured in triplicate using the automated Omron 705IT device (Omron, Kyoto, Japan) and following the manufacturer’s instructions. Adipose tissue and fasted blood samples were collected, processed and frozen at −80 °C for analysis at a later date. Women were also interviewed during the study visit and data were recorded on their level of education and various assets owned by their household.

### Study design

Participants attended two study visits at the MRCG@LSHTM facility in Fajara. During the first visit, gut permeability was assessed using the lactulose:mannitol (L:M) dual-sugar intestinal permeability test [18]. After an overnight fast and following complete bladder voiding, participants were given a 20 ml solution of two metabolically inert sugars, lactulose (Sandoz, Camberley, Surrey, UK) and mannitol (Sigma–Aldrich, Gillingham, Kent, UK) at a concentration of 200 g/L and 50 g/L respectively. Urine was collected for 5 h into containers containing three drops of chlorohexidine gluconate (5% w/v, Holden Medical, Lelystad, The Netherlands). The chlorohexidine gluconate was added to prevent bacterial contamination and sugar degradation. Participants were allowed to eat and drink 1 h after the L:M dose. At the end of collection urine aliquots were stored at −80 °C. The frozen samples were subsequently transported on dry ice to the MRC Elsie Widdowson Laboratory in Cambridge and stored at −80 °C until analysis.

During the second visit, participants were given a standardised 49 g fat load after an overnight fast. The meal, designed to match a typical composition of a traditional urban Gambian breakfast, was prepared with bread, salted butter and *Hellman’s Real Mayonnaise*. Of the total fat, 12.03 g were saturated fat, 25.45 g monounsaturated fatty acids and 11.48 g polyunsaturated fatty acids. Participants consumed only water and were not physically active during the test. Blood samples were collected at baseline (fasted) and at 2 h and 5 h post prandial. Aliquots of serum and plasma were stored at −80 °C until analysis. A needle-biopsy sample of subcutaneous adipose tissue was also obtained from the peri-umbilical area in fasted state as well as 5 h after ingesting the fat load. The adipose tissue samples were washed with saline, wrapped in aluminium foil, and snap frozen in liquid nitrogen prior to storing at −80 °C. These were subsequently transported on dry ice to the Oxford Centre for Diabetes, Endocrinology and Metabolism for gene expression analysis.

### Laboratory analysis

#### Lactulose and mannitol measurement

Lactulose and mannitol were measured by isocratic reversed-phase Ultra Performance Liquid Chromatography (UPLC) on a Waters ACQUITY UPLC® BEH Amide column and detection on an AB Sciex QTrap 4000 mass spectrometer as described elsewhere [19, 20].

#### Analyses of LPS, EndoCAb IgG, EndoCAb IgM, IL-6 and sCD14

Plasma LPS concentration was determined by HPLC/MS/MS using methods described elsewhere [21]. LPS concentration was calculated as “esterified 3-hydroxy-myristate” (esterified 3HM) concentration, this fatty acid being the most abundant 3-OH-fatty acid present in the lipid A moiety of LPS. For this purpose, we first determined the total 3HM concentration (after HCl hydrolysis step) and the non-esterified 3HM concentration (without hydrolysis step). Esterified 3HM concentration was finally calculated as “Total 3HM” concentration minus “non-esterified 3HM” concentration.

Endotoxin-core IgM and IgG antibodies (EndoCAb) were measured with a commercially available ready-to-use EndoCAb ELISA assay (Hycult Biotech, Uden, Netherlands) with detection limits of 0.05 MMU/ml and 0.13 GMU/ml, as per manufacturer instruction. IL-6 was measured using Thermo Fisher Scientific (Life Technologies) IL-6 high sensitivity coated ELISA with a detection limit of 0.03 pg/mL. sCD14 was measured using the Hycult biotech human sCD14 ELISA with a minimum detection limit of 1.56 ng/ml. LBP was measured using Hycult biotech human LBP ELISA with a minimum detection limit of 4.4 ng/ml.

#### Analyses of glucose, lipids (total cholesterol triglycerides, LDL, VLDL and HDL cholesterol)

Venous blood samples were collected and analysed for total cholesterol, very-low-density lipoprotein (VLDL), high-density lipoprotein (HDL) and triglycerides at the MRCG@LSHTM clinical laboratories using a centrifugal biochemical analyser (Cobas Fara, Roche, UK). The Friedewald formula was used in calculating the level of LDL.

#### Gene expression studies

Adipose tissue samples were firstly homogenised in TRIzol reagent, after which mRNA was extracted from the tissues, reversed transcribed, and quantitative PCR assays were performed as previously described [22]. The mRNA expression of lipopolysaccharide binding protein (LBP), IL-6, TNF-α, the macrophage markers CD11c and CD68, the activated macrophage (mannose receptor) marker CD206, leptin, peroxisome proliferator-activated receptor gamma (PPARG) and lipoprotein lipase (LPL) was normalised to three previously validated endogenous control genes: UBC, PPIA and PGK-1.

### Statistical analysis

Descriptive statistics (median, first and third quartiles) at baseline (fasted state) for variables such as age, anthropometric measures, blood pressure, heart rate and cholesterol levels were presented by study group. A composite index based on household’s assets was generated as a proxy indicator of household’s wealth using principal component analysis. The composite index was then divided in three groups (terciles) representing the poorest (1), middle (2) and richest (3) households. The non-parametric Kruskal–Wallis test was used to assess the similarity of the distribution of these baseline variables across study groups. Dunn’s test was performed for pairwise comparisons when there was evidence of difference between groups. Bonferroni’s adjustment was applied to control for type I error inflation due to multiple testing.

Outcome variables measured repeatedly (fasted baseline, 2 h and 5 h post prandially) such as esterified 3-hydroxymyristate (esterified 3HM), IL-6, EndoCAb IgM, EndoCAb IgG, sCD14 and lipopolysaccharide binding protein (LBP) were compared across study groups using a random-intercept linear regression model, adjusted for age (after log-transformation to adjust for positive skewness). We tested for interaction between group and time point, and performed pairwise comparisons between groups at each time point. Bonferroni’s adjustment was also applied to adjust for multiple testing. Age-adjusted geometric means and 95% confidence intervals were computed.

## Results

### Baseline characteristics

The baseline characteristics of the lean women, women with obesity and women with obesity-diabetes are shown in Table 1. Mean height was very similar across groups but, by design, the lean group were lighter and slimmer with lower fat and fat-free mass. The proportion of lean women were highest and lowest in the poorest and richest terciles respectively and vice versa for women with obesity-diabetes. Whilst availability of water within homes was similar between groups, the availability of flush toilets was highest for women with obesity-diabetes followed by those with obesity alone. Highest level of education attained was similar between groups. The women with obesity and women with obesity-diabetes were well matched for BMI, body fat and lean body mass. The women with obesity-diabetes were significantly older than the lean women and women with obesity. We recruited only women without diabetes into the lean and obese groups, hence fasting blood glucose (FBG) was not statistically different and within normal limits for these groups. FBG was significantly higher in women with obesity-diabetes group despite all of them being on treatment. Systolic blood pressure, pulse pressure and pulse rates were significantly higher among the women with obesity-diabetes compared to the other two groups. Triglycerides and VLDL were significantly higher in women with obesity-diabetes than either of the other two groups. Serum HDL was similar between the women with obesity and obesity-diabetes, and both were lower than the lean individuals.

**Table 1:** Baseline characteristics of the lean women, women with obesity and women with obesity-diabetes participating in the study.

Variables	Lean women (*n* = 49)	Women with obesity (*n* = 62)	Women with obesity-diabetes (*n* = 38)	Overall *P*	*P*^a^
Age (years), median (1st to 3rd quartiles)	37 (33–43)	38 (35–48)	51 (46–56)	<0.001^b^	<0.001
Education, *n* (%)				0.30^c^	–
None	26 (53.1)	24 (38.7)	13 (34.2)		
Lower basic	7 (14.3)	16 (25.8)	11 (29.0)		
Junior Secondary	9 (18.3)	8 (12.9)	4 (10.5)		
Senior secondary and above	7 (14.3)	14 (22.6)	10 (26.3)		
Water within the home, *n* (%)	38 (77.6)	58 (93.6)	32 (84.2)	0.05^c^	–
Flush toilet, *n* (%)	19 (38.8)	33 (53.2)	31 (81.6)	<0.001^c^	<0.001^d^
Wealth terciles, *n* (%)				0.002^c^	0.02^d^
1 (Poorest)	24 (49.0)	23 (37.1)	4 (10.6)		
2	16 (32.6)	25 (38.7)	17 (44.7)		
3 (Richest)	9 (18.4)	15 (24.2)	17 (44.7)		
Anthropometric parameters					
Height (m)	1.62 (1.60–1.66)	1.62 (1.59–1.65)	1.63 (1.58–1.66)	0.97^b^	–
Weight (kg)	53.6 (40.2–60.3)	85.3 (79.6–97.1)	88.3 (84.3–97.8)	<0.001^b^	0.61
BMI (kg/m^2^)	20.8 (19.3–22.2)	32.1 (30.7–35.8)	33.8 (32.2–36.3)	<0.001^b^	0.31
Fat (%)	28.8 (26.3–33.5)	45.3 (41.9–46.7)	45.6 (42.8–49.1)	<0.001^b^	0.69
Fat mass (kg)	15.0 (13.3–19.8)	38.4 (33.6–44.8)	40.7 (36.1–46.7)	<0.001^b^	0.55
Fat-free mass (kg)	38.2 (37.2–40.1)	47.3 (45.1–54.5)	49.9 (45.5–52.7)	<0.001^b^	0.72
FM index (kg/m^2^)	9.6 (8.1–12.1)	23.6 (20.6–27.0)	24.9 (22.1–28.7)	<0.001^b^	0.46
FFM index (kg/m^2^)	23.9 (23.0–24.7)	29.5 (28.1–31.5)	30.2 (28.7–32.4)	<0.001^b^	0.60
Cardiometabolic					
Fasted blood glucose (mmol/L)	4.9 (4.6–5.1)	5.2 (4.9–5.7)	9.4 (7.0–10.9)	<0.001^b^	<0.001
Systolic BP (mmHg)	115 (110–127)	127 (116–137)	137 (129–169)	<0.001^b^	0.004
Diastolic BP (mmHg)	75 (67–83)	81 (72–89)	84 (77–96)	<0.001^b^	0.12
Pulse pressure (mmHg)	42 (37–48)	47 (40–56)	55 (46–72)	<0.001^b^	0.003
Pulse rate (bpm)	74 (67–79)	69 (66–77)	80 (74–90)	<0.001^b^	<0.001
Cholesterol (mmol/L)	5.1 (4.5–5.4)	5.1 (4.4–5.6)	5.4 (4.4–6.1)	0.25^b^	–
Triglycerides (mmol/L)	0.71 (0.55–0.85)	0.90 (0.67–1.24)	1.31 (1.08–1.61)	<0.001^b^	0.002
LDL (mmol/L)	3.2 (2.4–3.7)	3.1 (2.9–4.1)	3.6 (2.6–4.2)	0.19^b^	–
HDL (mmol/L)	1.5 (1.3–1.7)	1.3 (1.1–1.4)	1.2 (1.1–1.4)	0.002^b^	0.61
VLDL (mmol/L)	0.32 (0.25–0.39)	0.41 (0.31–0.57)	0.60 (0.50–0.74)	<0.001^b^	0.002

*FM* fat mass, *FFM* fat-free mass, *BP* blood pressure, *LDL* low-density lipoprotein, *HDL* high-density lipoprotein, *VLDL* very-low-density lipoprotein.

^a^Dunn’s test comparing women with obesity and women with obesity-diabetes with Bonferroni correction.

^b^Fisher’s exact test.

^c^Kruskal–Wallis test.

^d^Fisher’s exact test comparing obese and obese-diabetic with Bonferroni correction.

### Gut permeability

**Fig. 1:**
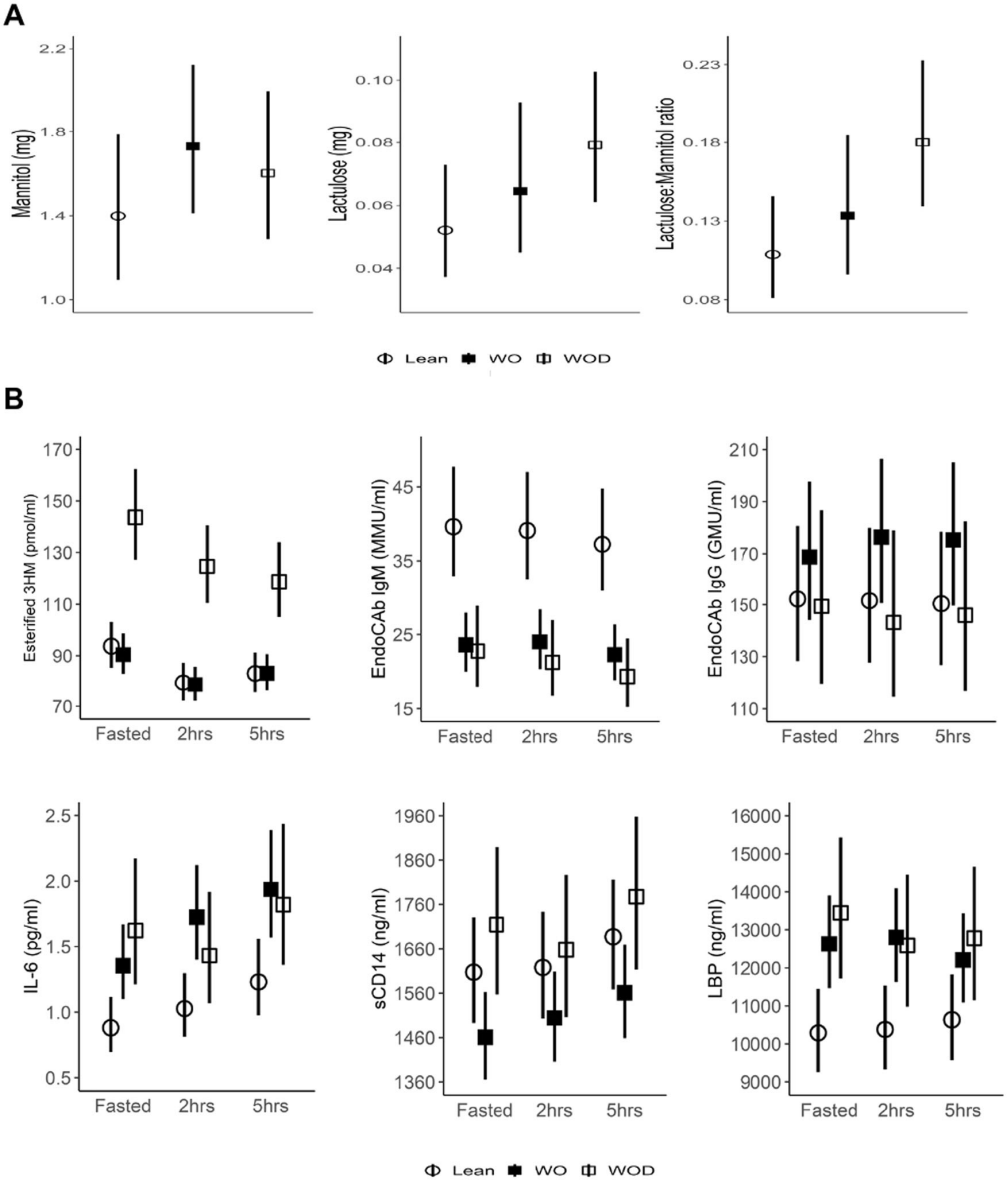
Gut permeability and markers of LPS translocation, sequestration and the inflammatory response. **A** Lactulose-mannitol assessment of gut permeability. Mannitol recovery assesses transcellular passage across enterocytes, lactulose recovery assesses paracellular passage and the lactulose-mannitol ratio assess permeability. **B** Predictive (age-adjusted) geometric mean of esterified 3HM and associated inflammatory and immunological markers at fasting, 2 h and 5 h post prandial. Plots shows geometric means and 95% confidence interval.

There was no difference in mannitol or lactulose excretion between the study groups, nor in the lactulose-mannitol ratio (Fig. 1A).

### Fasted and post-prandial changes in esterified 3HM and associated biomarkers

Figure 1B displays the age-adjusted geometric mean and 95% CI at each time point for esterified 3HM and associated inflammatory and immunological markers (see Table 2 for age-adjusted statistical comparisons; unadjusted statistical comparisons are found in supplementary Table 1). Esterified 3HM was much higher in women with obesity-diabetes than in lean women and women with obesity at all time points (*P* < 0.001), but there was no evidence for a difference between lean women and women with obesity. Unexpectedly, the level of esterified 3HM declined after the test meal in women with obesity-diabetes. IL-6 was lower in lean women compared to women with obesity and women with obesity-diabetes at fasting and remained so at 2 h and 5 h post prandial (*P* = 0.003), with the level of IL-6 increasing over time particularly in the lean women and women with obesity. IL-6 levels did not differ between women with obesity and women with obesity-diabetes.

**Fig. 2:**
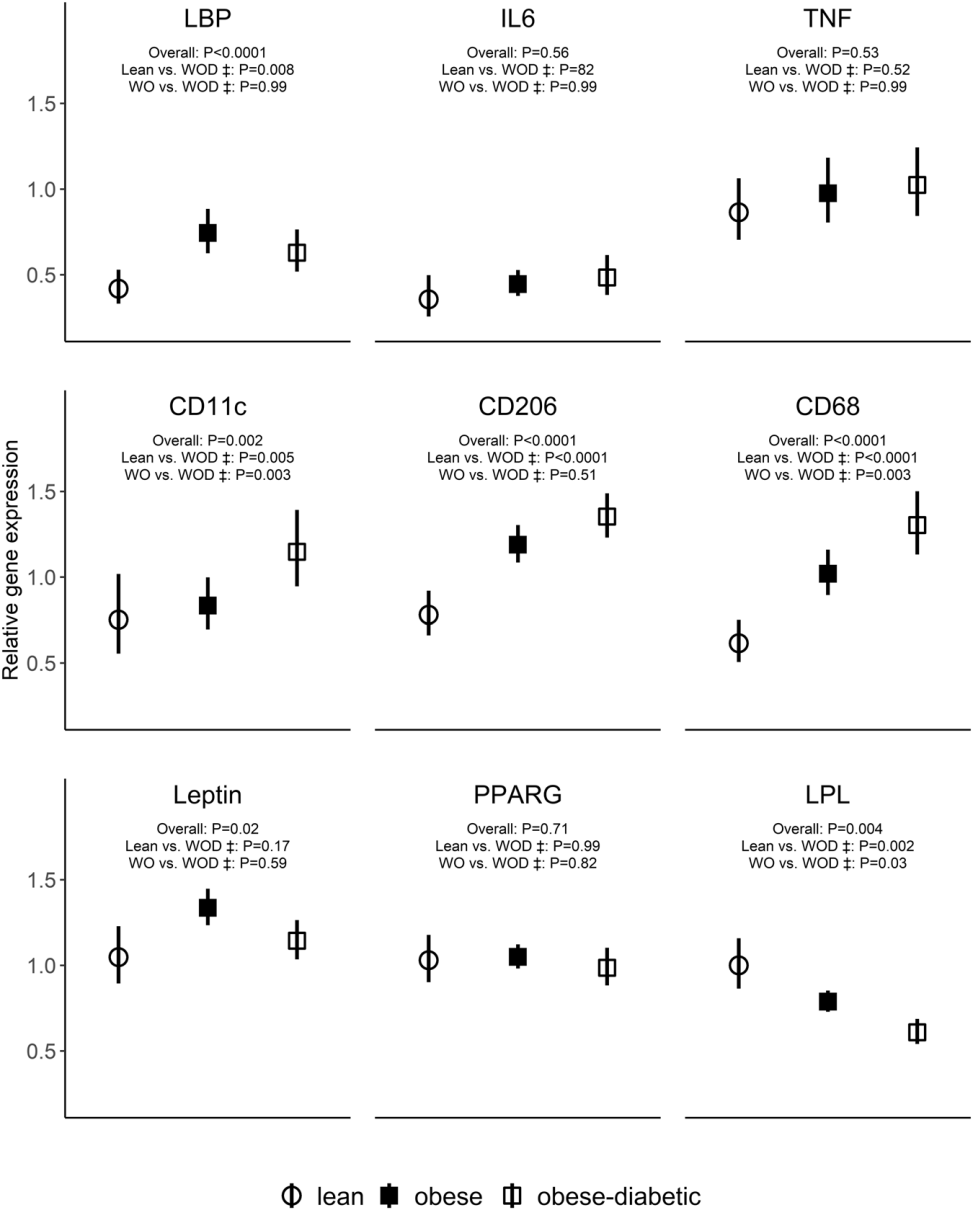
Relative gene expression in subcutaneous adipose tissue biopsy. LBP lipopolysaccharide binding protein, IL-6 interleukin-6, TNF tumour necrosis factor-α, PPARG Peroxisome Proliferator-Activated Receptor Gamma, LPL lipoprotein lipase, CD11c is a marker for monocytes and macrophages; CD206 (macrophage mannose receptor) is a marker for alternatively activated macrophages; CD68 is a marker for tissue macrophages; WO women with obesity, WOD women with obesity and diabetes.

**Table 2:** Predictive (age-adjusted) geometric means by group and time point.

Marker	Time	Geometric mean (95% CI)			*P*^a^	*P*^b^	*P*^c^	*P*^d^
		Lean women	Women with obesity	Women with obesity-diabetes				
Esterified 3HM (pmol/ml)	Fasting	94 (85–103)	90 (83–99)	144 (127–162)	<0.001	0.99	<0.001	0.34
	2 h	79 (72–87)	79 (72–86)	125 (111–141)		0.99	<0.001	
	5 h	83 (76–91)	83 (76–91)	119 (105–134)		0.99	<0.001	
IL-6 (pg/ml)	Fasting	0.88 (0.70–1.12)	1.36 (1.10–1.67)	1.62 (1.21–2.17)	0.003	0.04	0.99	0.03
	2 h	1.03 (0.81–1.30)	1.72 (1.40–2.12)	1.43 (1.07–1.92)		0.006	0.99	
	5 h	1.23 (0.97–1.56)	1.94 (1.57–2.39)	1.82 (1.36–2.44)		0.02	0.99	
EndoCAb IgM (MMU/ml)	Fasting	40 (33–48)	24 (20–28)	23 (18–29)	<0.001	<0.001	0.99	0.78
	2 h	39 (33–47)	24 (20–28)	21 (17–27)		<0.001	0.99	
	5 h	37 (31–48)	22 (19–26)	19 (15–24)		<0.001	0.99	
EndoCAb IgG (GMU/ml)	Fasting	152 (128–181)	169 (144–198)	149 (120–187)	0.35	NS	NS	0.40
	2 h	152 (128–180)	176 (151–206)	143 (115–179)		NS	NS	
	5 h	150 (127–178)	175 (150–205)	146 (117–182)		NS	NS	
sCD14 (ng/ml)	Fasting	1607 (1493–1731)	1460 (1365–1563)	1715 (1557–1889)	0.04	0.35	0.06	0.70
	2 h	1618 (1502–1743)	1504 (1406–1609)	1658 (1506–1826)		0.87	0.68	
	5 h	1687 (1568–1816)	1561 (1459–1670)	1777 (1614–1958)		0.71	0.21	
LBP (ng/ml)	Fasting	10293 (9254–11448)	12,628 (11,470–13,903)	13,446 (11,720–15,425)	0.01	0.03	0.99	0.003
	2 h	10380 (9335–11,541)	12,802 (11,629–14,093)	12,591 (10,976–14,445)		0.02	0.99	
	5 h	10,639 (9569–11,828)	12,203 (11,087–13,432)	12,782 (11,142–14,664)		0.33	0.99	

*3HM* 3-hydroxy-myristate, *IL-6* interleukin-6, *EndoCAb* endotoxin-core antibody, *sCD14* soluble CD14, *LBP* lipopolysaccharide binding protein.

^a^Overall test of difference between groups with Bonferroni correction.

^b^Test of difference between lean women and women with obesity by time point with Bonferroni correction.

^c^Test of difference between women with obesity and women with obesity-diabetes by time point with Bonferroni correction.

^d^Test for interaction between group and time point.

The level of EndoCAb IgM was much higher in lean women than women with obesity and women with obesity-diabetes at all time points (*P* < 0.001), but there was no evidence of a difference between women with obesity and women with obesity-diabetes. A slight decline was noted over time across groups.

EndoCAb IgG and sCD14 levels did not differ across groups. There was evidence of a difference in LBP levels across groups (*P* = 0.01). Lean women had lower LBP level compared to women with obesity and women with obesity-diabetes (*P* = 0.03). There was strong evidence that the differences across groups changed with time (*P* = 0.003).

There was generally a weak correlation between biomarkers in each of the study groups and among study participants combined for all the respective time points. These are shown in Supplementary Fig. 1.

### Relative gene expression in AT

We further explored gene expression for markers of inflammation and macrophage function as well as markers of fat metabolism in AT (Fig. 2). (i) *Inflammation*: mRNA levels for the inflammatory markers IL-6 and TNF-α did not differ across the three study groups. LBP mRNA was more highly expressed in individuals with obesity, regardless of diabetic status compared to lean individuals. (ii) *Macrophage function*: mRNA levels for all markers of macrophage presence and function (CD11c, CD206 and CD68) showed a significant gradient across the lean, obese and obese-diabetic groups. CD206 expression was similar between the obese and obese-diabetic groups, but the women with obesity-diabetes had higher expression levels of CD11c and CD68 than their obese counterparts without diabetes. (iii) *Fat metabolism*: Surprisingly, leptin and PPARG expression were similar across the different study groups. LPL was more highly expressed in lean individuals compared to the other 2 groups.

## Discussion

This study examined two hypothesised mechanisms to explain the raised levels of LPS responsible for ME in women with obesity-diabetes; paracellular transfer across a leaky gut, or enhanced fat-mediated post-prandial translocation. Despite large differences in fat mass and fasting glucose levels between the study groups, and confirmation of the anticipated elevation of LPS in women with obesity-diabetes, we found no evidence to support either mechanism. A possible explanation is that translocation rates of pro-inflammatory bacterial debris are similar across the experimental groups, but that women with obesity and women with obesity-diabetes have a lower ability to sequester and detoxify LPS and related compounds. The markedly lower levels of EndoCAb IgM in women with obesity and obesity-diabetes respectively (confirming data from our prior study [8]) would support this hypothesis.

Evidence suggesting that a breakdown in gut integrity leads to the translocation of bacterial debris with consequent systemic and tissue-specific inflammation largely derives from animal studies (reviewed by Massier [3]). Loss of gut barrier integrity in *ob/ob* and *db/db* obese mice was first demonstrated using ex vivo (Ussing chamber) analysis [23]. Cani’s group has further examined several aspects of the relationship between the gut microbiota and permeability in mice [24, 25]. Thais et al. show that intestinal barrier integrity in diabetic mice is compromised by hyperglycaemia through GLUT-2 transcriptional reprogramming of intestinal epithelial cells [15]. However, the evidence from human studies is equivocal. Using the sucralose/mannitol (SM) ratio Gummesson et al. reported a correlation between SM ratio and visceral fat mass [26]. Teixera *et al* reported a marginally higher lactulose secretion in 20 subjects with obesity than 20 lean, but there was no difference in the lactulose/mannitol (LM) ratio [27]. Damms-Machado et al. showed a decrease in LM ratio with weight loss in patients with severe obesity with steatosis [28]. This study is often cited as evidence for an association between LM ratio and the HOMA index but the association was not significant at *P* < 0.05. As cited above, the mouse studies by Thaiss et al. [15] provide strong evidence that gut damage can be caused by hyperglycaemia, however, their human data is weak. Using circulating concentrations of pattern recognition receptor (PRR) ligands as a proxy for intestinal integrity in 29 subjects they reported a marginal correlation with HbA1c levels, but not with measures of fat mass, and furthermore the correlations with other variables such as height and red blood cells were almost as strong [15]. Thus, although frequently cited, a closer examination of the human evidence linking gut permeability to obesity and/or diabetes is highly questionable. Our study showed wide ranges in the LM ratio within each group but there is no evidence for a difference in gut integrity between the lean women, women with obesity and women with obesity-diabetes. Note that our prior study also revealed no difference in faecal calprotectin levels across groups [8].

In our prior study we used the limulus amebocyte lysate (LAL) assay for endotoxin quantitation [8]. In the current study we measured esterified 3HM by mass spectrometry as a more precise and direct assay for LPS [21]. In both studies observations were consistent with endotoxin/LPS which was very significantly raised in the patients with diabetes (+35%, *P* = 0.02 in Study 1 and +56%, *P* < 0.001 here) but not in the obese controls which, in both studies, showed virtually identical levels to their lean counterparts. Contrary to our hypothesis that there might be a chylomicron-mediated surge in LPS following consumption of the high-fat test meal there was in fact a 17% decline in LPS by 5 hours in the individuals with obesity-diabetes and no perceptible change in the other two groups. Pendyala et al. observed a 71% increase in plasma LPS activity with Western diet and a reduction by 31% following the introduction of a prudent diet in 8 healthy volunteers over a period of 1 month in a cross-over study [29]. Harte et al. observed a significant post-prandial increase in serum LPS levels in individuals with diabetes, impaired fasting glucose and subjects with obesity over a 4-hour period, but not in non-obese controls [11]. Clemente-Postigo et al. [30] also reported an acute rise in LPS following an oral fat load. The higher baseline serum LPS level in subjects with diabetes compared to others is a consistent finding across all studies, but the contrasting post-prandial responses require explanation. Harte et al. used a 75 g fat load whilst we and Clemente-Postigo et al. used 50 g. A more likely explanation may be the difference in methods for LPS quantification. Most studies have used the LAL test to assess endotoxemia. This test reflect biological activity of LPS which may not accurately reflect total LPS mass concentration, and has indeterminate interlaboratory variability as evidenced by very different LPS concentrations between studies [31]. Furthermore, LAL is only restricted to the active LPS fraction and does not give account of the total amount of LPS. The direct quantification of plasma LPS using HPLC/MS/MS, as in this study, gives a more accurate reflection of total LPS in plasma. Under normal conditions LPS is effectively cleared from the circulation by hepatocytes which recognise and remove bacterial products from the circulation. There is evidence from elsewhere that this process is defective in patients with diabetes due to hyperglycaemia and hyperinsulinemia which compromise macrophage and monocyte function [13]. However, the post-prandial decline in LPS in our data reveals that active clearance is occurring, possibly accelerated by the presence of post-prandial lipaemia, albeit against a higher chronic background level of LPS.

Of the biomarkers we have studied, IL-6 is to the best of our knowledge the only to have been assessed among healthy volunteers at population level. A recent meta-analysis including 57 studies of 3166 healthy volunteers reported a pooled IL-6 mean estimate of 5.2 pg/ml (95% CI: 4.6–5.7) varying between 0 and 43.5 pg/ml [32]. The mean obtained in our study sample was however significantly lower being 0.86 pg/ml (0.69–1.08) in lean women, 1.33 pg/ml (1.08–1.63) in women with obesity and 1.70 pg/ml (1.31–2.20) in women with obesity-diabetes (Supplementary Table 1). This gradient increase across the 3 groups from lean women to women with obesity-diabetes closely replicate our prior study [8]. All groups showed a post-prandial increase in IL-6. In contrast, levels of the monocyte activation marker sCD14 (not assayed in our previous study) did not differ between groups and showed no significant change following the meal.

We measured three markers of LPS binding; LBP, and EndoCAb IgG and IgM antibodies. None of these showed any acute post-prandial changes but, with the exception of EndoCAb IgG which was similar across all groups, there were notable differences in levels of these binding markers between the groups at all time points. LBP (not measured in our prior study) was higher in the two groups with obesity and EndoCAb IgM was notably lower. This profound and paradoxical reduction in EndoCAb IgM is strongly replicated across both our studies.

Working on the principle that fat soluble LPS might accumulate in adipose tissue leading to macrophage invasion and activation [33, 34] we assayed RNA expression levels in peri-umbilical subcutaneous adipose tissue biopsies. Expression levels of surface markers for macrophage infiltration were raised in obese individuals whether or not they were diabetic. This is consistent with findings from previous studies [35]. Both the marker for total macrophage infiltration (CD68) and its pro-inflammatory counterpart (CD11c) showed similar trends with both being most highly expressed in women with obesity-diabetes and significantly least expressed in lean populations. These markers have been causally linked to insulin resistance [35], hence consistent with our findings. The anti-inflammatory marker (CD206) was also similar in relative expression but with no significant difference between the groups with obesity. Although surprising, this was consistent with a report by Jia *et al* where the level of CD206 was positively correlated with insulin resistance indices [36]. Recent studies on human adipose tissue have corroborated that macrophages may express both pro- and anti-inflammatory markers as part of their inflammatory response [36, 37].

Leptin expression, which we expected to be higher in obese groups, was similar across groups. Since our levels were normalised against standard housekeeping genes the raised levels of circulating leptin in obesity may result from the greater adipocyte number rather than an increase per adipocyte. Leptin expression is also known to be influenced by the presence of ovarian sex steroids levels of which are expected to be higher in the younger premenopausal groups without diabetes [38]. PPARG was also similarly expressed in adipose tissue in the three study groups, suggesting similar capacity of adipose tissue to store lipid among other functions. We expected this to be highest in the lean group and lowest in the group with obesity-diabetes as has been previously suggested [39]. Our data showed significantly lower expression of LPL in participants with obesity especially among those with diabetes. This enzyme is essential in hydrolysis of triglycerides and very-low-density lipoprotein [40], which perhaps explains at least in part the serum levels of these in those with diabetes relative to the other groups without diabetes.

Our study has some limitations. We only measured small intestinal permeability using the notably variable L:M ratio; however, there was not even a suggestion of any difference between the study groups. We also conducted the LM test at a single time point, and an average of multiple tests could have been a more accurate assessment. There was a large age difference between the group with obesity- diabetes and the other two groups, with the former including mainly peri-menopausal women. This may have confounded our findings; for instance it is quite possible that the differences noted between the women with obesity and women with obesity-diabetes reflect the stage of disease development, rather than indicating differences between diabetes susceptible and non-susceptible individuals. Additionally, the quantity of fat given to study participants may have been sub-optimal to trigger acute inflammatory and immunological responses. It remains possible that the raised levels of LPS in subjects with obesity and obesity-diabetes are the result of long-term ingestion of high-fat diets; effects which we did not assess in the present study.

In summary, we have robustly confirmed that women with obesity-diabetes have raised circulating levels of gut-derived bacterial debris (indicated by LPS) but our data refute the hypothesised mechanisms of a leaky gut or enhanced post-prandial LPS translocation in chylomicrons. Our striking confirmation of much lower levels of EndoCAb IgM antibodies in women with obesity and women with obesity-diabetes suggest an explanation analogous to, and parallel with, the development of glucose intolerance. In the early phases of obesity-induced pathology, glucose sensitivity is maintained by hyperinsulinaemia, leading to pancreatic beta-cell exhaustion. Likewise, translocated LPS may be successfully detoxified and eliminated in the early phases of disease but then lead to exhaustion of the EndoCAb IgM neutralising capacity which allows a build-up of adipose tissue LPS, attraction and activation of macrophages, and the consequent downstream impairment of insulin action described elsewhere [33, 34]. Further elucidation of the drivers of bacterial translocation (especially the study of the chronic vs acute effects of high-fat diets) may help refine future dietary interventions.

## Supplementary information

Supplementary Appendix

Supplementary Figure 1

Supplementary Figure 2

## Data Availability

The datasets generated during and/or analysed during the current study are available from the corresponding author on reasonable request.
