# Supplementary material for: Possible mediators of metabolic endotoxemia in women with obesity and women with obesity-diabetes in The Gambia
Source: Int J Obes (Lond). 2022 Aug 6;46(10):1892–900. doi: 10.1038/s41366-022-01193-1 (PMC9492538; doi:10.1038/s41366-022-01193-1)
Supplement: Supplementary file 1 — Supplementary Appendix [file 41366_2022_1193_MOESM1_ESM.docx]

Supplementary Appendix

Supplementary Figure 1: Correlation between biomarkers in each of the study groups and among study participants combined

Supplementary Figure 2: Scatter plots of biomarkers in each of the study groups and among study participants combined

Supplementary Table 1: Geometric means (unadjusted) by group and time point

| Marker | Time | Geometric mean (95% CI) | | | P^a^ | P^b^ | P^c^ | P^d^ |
| --- | --- | --- | --- | --- | --- | --- | --- | --- |
|  |  | Lean | Obese | Obese-diabetic |  |  |  |  |
| Esterified 3HM (pmol/ml) | Fasting | 90  (82-99) | 88  (81-97) | 158  (141-177) | <0.001 | 0.99 | <0.001 | 0.31 |
|  | 2 hours | 75  (69-84) | 77  (70-84) | 137  (123-154) |  | 0.99 | <0.001 |  |
|  | 5 hours | 80  (72-88) | 81  (75-89) | 131  (117-146) |  | 0.99 | <0.001 |  |
| IL-6 (pg/ml) | Fasting | 0.86  (0.69-1.08) | 1.33  (1.08-1.63) | 1.70  (1.31-2.20) | <0.001 | 0.03 | 0.87 | 0.03 |
|  | 2 hours | 1.01  (0.80-1.26) | 1.68  (1.37-2.06) | 1.50  (1.15-1.94) |  | 0.005 | 0.99 |  |
|  | 5 hours | 1.21  (0.96-1.51) | 1.89  (1.54-2.32) | 1.90  (1.47-2.47) |  | 0.02 | 0.99 |  |
| EndoCAb  IgM (MMU/ml) | Fasting | 43  (36-52) | 24  (20-29) | 19  (15-24) | <0.001 | <0.001 | 0.56 | 0.78 |
|  | 2 hours | 42  (35-51) | 25  (21-29) | 18  (14-22) |  | <0.001 | 0.13 |  |
|  | 5 hours | 40  (34-48) | 23  (19-27) | 16  (13-20) |  | <0.001 | 0.09 |  |
| EndoCAb  IgG (GMU/ml) | Fasting | 145  (123-171) | 165  (141-193) | 166  (136-203) | 0.35 | NS | NS | 0.40 |
|  | 2 hours | 144  (122-171) | 172  (148-201) | 159  (131-194) |  | NS | NS |  |
|  | 5 hours | 143  (121-169) | 172  (147-200) | 162  (133-198) |  | NS | NS |  |
| sCD14 (ng/ml) | Fasting | 1572  (1461-1692) | 1440  (1345-1541) | 1809  (1655-1976) | <0.001 | 0.50 | <0.001 | 0.74 |
|  | 2 hours | 1582  (1470-1702) | 1476  (1380-1579) | 1748  (1600-1910) |  | 0.99 | 0.02 |  |
|  | 5 hours | 1650  (1535-1774) | 1540  (1440-1647) | 1874  (1715-2048) |  | 0.99 | 0.003 |  |
| LBP (ng/ml) | Fasting | 10410  (9397-11533) | 12798  (11650-14059) | 13117  (11602-14831) | 0.01 | 0.02 | 0.99 | 0.002 |
|  | 2 hours | 10498  (9479-11628) | 12984  (11822-14260) | 12284  (10865-13889) |  | 0.02 | 0.99 |  |
|  | 5 hours | 10761  (9117-11917) | 12365  (11260-13579) | 12470  (11029-14099) |  | 0.29 | 0.99 |  |

Overall test of difference between groups. ^b^ Test of difference between lean and obese by time point with Bonferroni correction. ^c^ Test of difference between obese and obese-diabetic by time point with Bonferroni correction. ^d^ Test for interaction between group and time point. Abbreviations: 3HM, 3-hydroxy-myristate; IL-6, interleukin-6; EndoCAb, endotoxin core antibody; sCD14, soluble CD14; LBP, lipopolysaccharide binding protein
