# Supplementary material for: Possible mediators of metabolic endotoxemia in women with obesity and women with obesity-diabetes in The Gambia
Source: Int J Obes (Lond). 2022 Aug 6;46(10):1892–900. doi: 10.1038/s41366-022-01193-1 (PMC9492538; doi:10.1038/s41366-022-01193-1)

A Fasted (baseline)

All groups combined

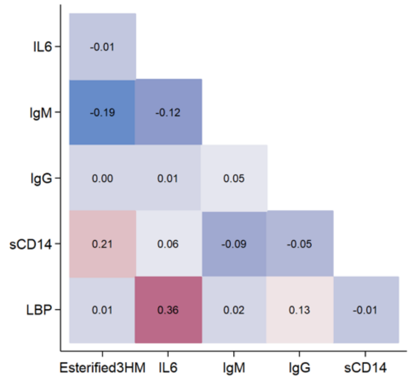

Lean women

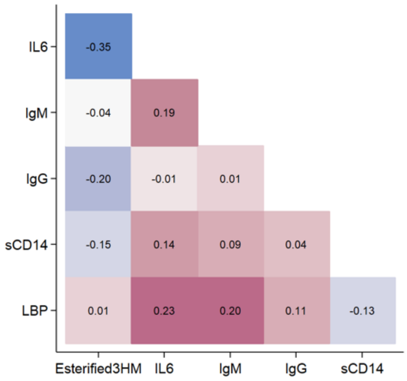

Women with obesity

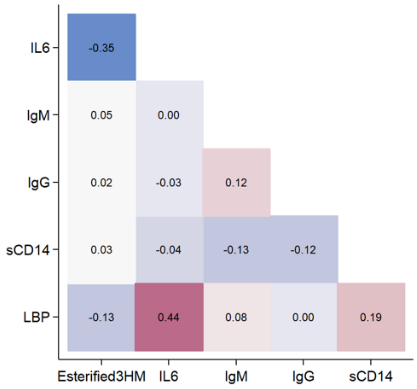

Women with obesity-diabetes

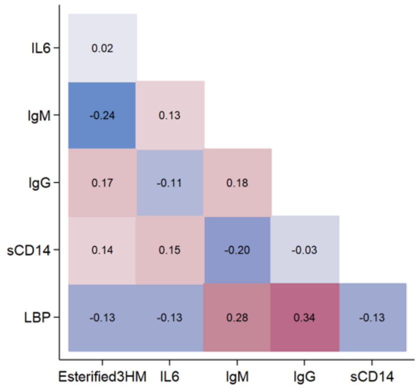

B 2 hours postprandial

All groups combined

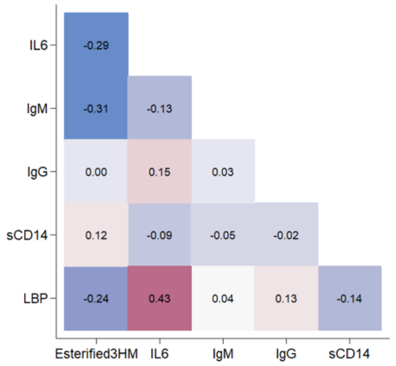

Lean women

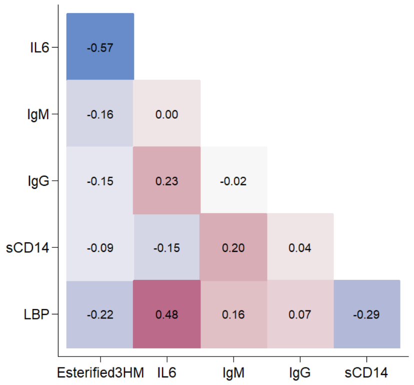

Women with obesity

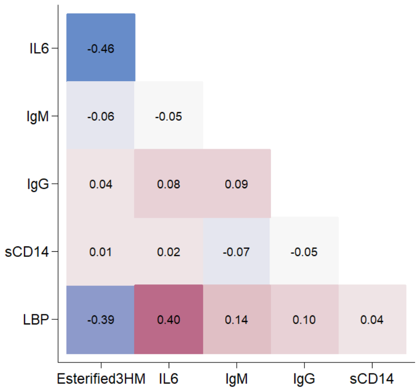

Women with obesity-diabetes

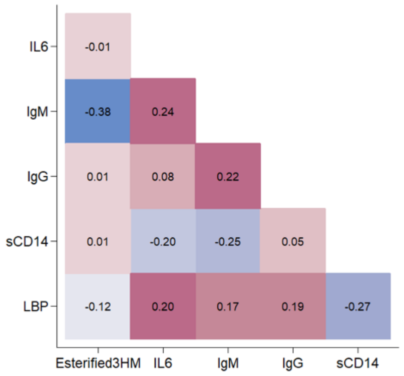

C 5 hours postprandial

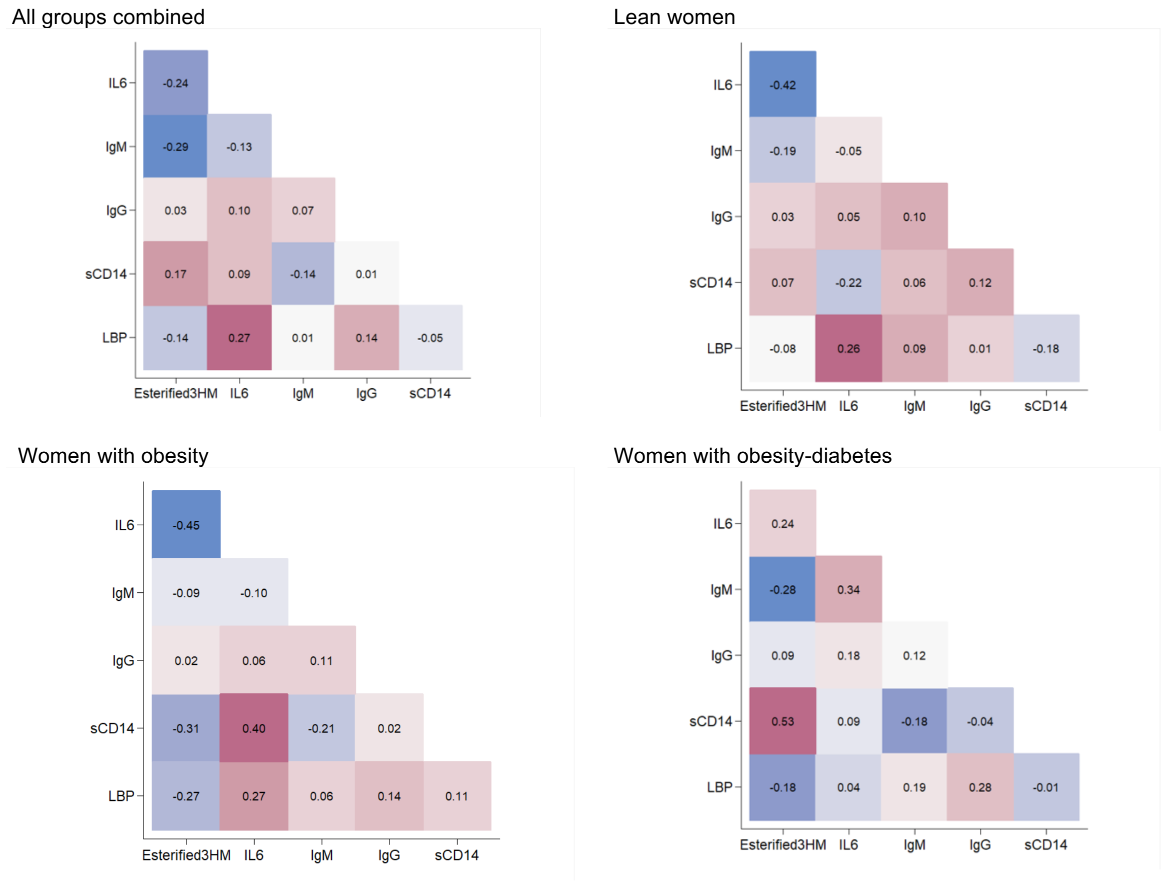

Supplement: Supplementary file 2 — Supplementary Figure 1 [file 41366_2022_1193_MOESM2_ESM.pdf]
